# Components of Implicit Stigma against Mental Illness among Chinese Students

**DOI:** 10.1371/journal.pone.0046016

**Published:** 2012-09-28

**Authors:** Xiaogang Wang, Xiting Huang, Todd Jackson, Ruijun Chen

**Affiliations:** 1 Key Laboratory of Cognition and Personality, Southwest University, Chongqing, China; 2 Faculty of Psychology, Southwest University, Chongqing, China; 3 Sichuan Judicial and Police Officers Professional College, Deyang, China; 4 School Culture and Social Development Studies, Southwest University, Chongqing, China; 5 College of Education Science, Zhengzhou Normal University, Zhengzhou, China; Federal University of Rio de Janeiro, Brazil

## Abstract

Although some research has examined negative automatic aspects of attitudes toward mental illness via relatively indirect measures among Western samples, it is unclear whether negative attitudes can be automatically activated in individuals from non-Western countries. This study attempted to validate results from Western samples with Chinese college students. We first examined the three-component model of implicit stigma (negative cognition, negative affect, and discriminatory tendencies) toward mental illness with the Single Category Implicit Association Test (SC-IAT). We also explored the relationship between explicit and implicit stigma among 56 Chinese university college students. In the three separate SC-IATs and the combined SC-IAT, automatic associations between mental illness and negative descriptors were stronger relative to those with positive descriptors and the implicit effect of cognitive and affective SC-IATs were significant. Explicit and implicit measures of stigma toward mental illness were unrelated. In our sample, women's overall attitudes toward mental illness were more negative than men's were, but no gender differences were found for explicit measures. These findings suggested that implicit stigma toward mental illness exists in Chinese students, and provide some support for the three-component model of implicit stigma toward mental illness. Future studies that focus on automatic components of stigmatization and stigma-reduction in China are warranted.

## Introduction

Stigmatization of mental illness remains a serious social issue, occurring across nations and cultures worldwide [1], [2]. It is a significant barrier to the seeking of mental health service, successful treatment and rehabilitation [3], [4]. Since overt prejudice toward people with mental illness has become less acceptable, indirect expression of negative attitudes may be more common. A recent review by Stier and Hinshaw that summarized the previous stigma studies indicated that self-reported, explicit attitude measures were problematic [5]; explicit measures are likely to underestimate the true levels of stigma because they are subject to social desirability biases [3], [6], [7]. In addition, explicit attitudes of the general public toward people with mental illness have been found to be more positive than implicit attitudes [8]. Teachman et al also found a low level of explicit bias toward mental illness on semantic differential scales relative to the assessment of automatic negative attitudes [7].

Researchers have placed more interest on automatic attitudes toward people with mental illness to better understand mental illness stigma [7], [10], [9], [11]. The pioneering study of Teachman et al found that the central components of stigma may be activated in an automatic, implicit manner that is not associated with explicit evaluation [7]. A series of studies have shed more light on the automatic cognitive and emotional aspects of mental illness stigma by using the Implicit Association Test (IAT) or the Brief Implicit Association Test (BIAT) [10]–[12]. They found that research participants tend to automatically associate mental illness with negative words (e.g., bad, guilt, and dangerous) rather than with positive words (e.g., good, innocent, and competent) [3], [6]. The key emotional aspects of implicit stigma, which focuses on “anger” and “shame,” have also been identified [8], [10].

Despite the above evidence of implicit attitudes toward mental illness, some questions remain about the components of implicit stigma against mental illness. In reality, mental illness stigma is one's unfavorable attitude toward mental illness [13]. The Affect-Behavior-Cognition theory of attitudes indicates that a favorable or unfavorable evaluative reaction toward someone exhibited in one's beliefs, feelings, or intended behavior. More recent conceptions of stigma have also considered three components of stigma toward mental illness [1], [2], [7]: (1) cognitive evaluations, which are knowledge structures or beliefs about individuals with mental illness; (2) affective reactions, which reflect emotional components; and (3) discrimination, which are negative actions against the group. In addition, Xu and Zhang used event-related brain potentials to investigate implicit attitudes toward smokers in neural processing and found some evidences supporting the Affect-Behavior-Cognition theory of attitudes [14]. To date, research on mental illness stigma has largely focused on the cognitive and affective aspects [8], [10], [11] while the question of whether implicit stigma toward mental illness has automatically activated behavioral tendencies has been neglected. Thus, we proposed that implicit stigma toward mental illness, which potentially mediates one's beliefs and reactions toward people with mental illness, includes automatically activated negative cognition, spontaneously activated affective reaction and discriminatory tendencies.

The IAT and BIAT are the most commonly used procedures to assess implicit stigma [3], [6], [8], [10]. Both use complementary pairs of concepts and attributes. These measurement techniques are limited to measuring the relative strengths of pairs of associations rather than absolute strengths of single associations [15]. However, it is noteworthy that there is no natural complementary pair for “mental illness,” in contrast to implicit evaluations of gender (female-male) and race (black-white) biases. Researchers have typically considered mental illness as a generally negative concept that reflects illness, resulting in the use of attitudes toward physical illness are comparisons [7]. In cases wherein attitudes toward mental illness are assessed relative to physical illness, the comparisons cannot absolutely or completely reflect the association of mental illness with “bad” or “good.” It is possible, however, to directly assess automatic, implicit stigma toward people with mental illness. Several implicit measures have been developed to assess evaluative associations with a single target, for example, the Single Category Implicit Association Test (SC-IAT) [16], the Go/No-Go Association Task [17], and priming-based measures [18].

Previous studies have also found inter-cultural variations in the explicit stigma attached to mental illness [1], [19]. Therefore, it is necessary to examine the evidence of implicit stigma in under-studied non-Western countries. Recent research in Chinese societies, including those in mainland China [20], Hong Kong [21], and Singapore [22], has identified stigma as a prominent factor that has negative effects on individuals with mental illness. The role of cultural context in shaping mental illness stigma was evident. Yang proposed that the particular manifestations of the stigma associated with mental illness were shaped by cultural meaning embedded within Confucianism, the centrality of “face,” and the pejorative etiological beliefs of mental illness in Chinese societies [23]. Although mental illness stigma has been measured using a variety of self-reported measurements, few studies have investigated the components and prevalence of implicit stigma toward mental illness in Chinese samples [24]. Therefore, the present study aimed to examine the components and prevalence of implicit stigma toward mental illness using the SC-IAT with a Chinese sample and to compare the results with previous findings of implicit stigma; these aims were achieved.

## Methods

### Ethics Statement

The Ethics Committee of Southwest University granted ethical approval for the study procedures. All participants provided verbal consent prior to participation. They were assured that no harm would come to them in the process of experiment, and were told that this experiment involved three sorting tasks and two scales. The results of all tests were kept private. Participants were informed that they had the right to quit at any time during the experiment. Written consent were obtained before experiment began. All participants were paid (about 2.5 US dollars) and thanked after the experiment.

### Participants

We recruited the participants via study advertisements posted in the Bulletin Board System of Southwest University. Fifty-six healthy right-handed undergraduate students (29 female, 27 male) were recruited. All had normal or corrected-to-normal vision and no history of seeking professional psychological services. Their mean age was 20.42 years (SD = 1.23, range = 19–26); 91% were Han Chinese, and the rest were from other ethnic groups (Table 1).

**Table 1:** Demographic variables and the explicit measures among the sample.

	Measures	T	P-value
Age (years; *M*, SD)	20.42(1.23)	–	–
Gender (Female; n, %,)	29(52%)	–	–
Race (Han and Ethnic minority; n, %)	51(91%), 5(9%)	–	–
Social Distancec1 (scale-midpoint: 2.5, range 1–4)	11.18	−2.73	<0.01
Feeling Thermometer2 (scale-midpoint: 50, range 1–100 )	51.02	0.38	0.71

### Implicit measures

We used a computer-based response-latency measure, the SC-IAT, to assess automatic aspects of implicit stigma toward mental illness. This modification of the IAT has adequate psychometric properties [16]. Unlike the IAT and BIAT, in the SC-IAT, participants classify a series of words into three categories, namely, the target category (mental illness) and two attribute categories (bad versus good). In the current study, the target category label was “mental illness,” and the attribute category labels were “negative words” and “positive words.” The task requires items to be classified when the category labels are paired on either side of the screen. This paradigm assumed that the stimuli would be classified more quickly if the target and attribute category pairs match participants' automatic associations, and that the stimuli would be processed more slowly if these pairs contradict automatic associations.

In this study, the SC-IAT consisted of two stages. Each stage consisted of 24 practice trials that were immediately followed by 72 test trials. The three category labels were shown on either side of the screen at the top (i.e., “mental illness” or “negative words” on the right side, and “positive words” on the left side in one stage), and the stimuli words were presented at the center of the screen consecutively. In the first stage, participants were required to press a left-hand response key (*D*) if one stimuli word belonged to either “mental illness” or “negative words” and a right-hand response key (*F*) if it belonged to “positive words.” In order to preempt response bias, mental illness words, negative words, and positive words were presented in a 1∶1∶2 ratio such that the corrected response were split evenly between the *D* and *F* key. In the second stage, the participants' task was to press a left-hand response key (*D*) if the word belonged to “negative words” and a right-hand response key (*F*) if it belonged to either “mental illness” or “positive words.” Mental illness words, negative words, and positive words were presented in a 1∶2∶1 ratio in this stage. Each stage was preceded by a set of instructions concerning the dimensions of the sorting task and the appropriate key responses. Stimuli words appeared centered on the screen. Category reminder labels were appropriately positioned on the top of the screen. Each incorrect response was signaled by a red *X* centered below the stimulus for 150 ms, while correct response was signaled by a green *O* centered below the stimulus for 150 ms; this feedback differs from that of the IAT and the BIAT.

The three SC-IATs (*cognitive* SC-IAT, *affective* SC-IAT, and *behavioral* SC-IAT) were developed based on the postulated three components of implicit stigma toward mental illness to measure automatic aspects of stigma. The target category comprised the four most familiar mental illnesses from the pilot study. Out pilot study also used three steps to obtain valid attribute categories or words that reflect the components of implicit stigma. First, 173 informants were surveyed to write down as many words reflecting their reactions toward people with mental illness on the paper as possible. One hundred and twenty-six words consisting of two Chinese characters each (e.g., *ke-ai*, *hai-pa*, *tao-bi*) were captured in this investigation. Second, another group of informants (N = 40) sorted these words into three categories: *cognitive evaluation*, *affective reaction*, and *behavioral reaction*. Only the words that were sorted into the same category by 50% or more of the participants entered the third step: 35 words were categorized into *cognitive evaluation*, 31 into *affective reaction*, and 33 into *behavioral tendency*. Third, three groups of informants separately rated each word for valence (*N* = 78; on a 7-point scale: 1 = *definitely negative*, 7 = *definitely positive*), familiarity (*N* = 82; on a 5-point scale: 1 = *definitely unfamiliar*, 5 = *definitely familiar*), and meaningfulness (*N* = 82; on a 5-point scale: 1 = *definitely unmeaningful*, 5 = *definitely meaningful*). After balancing familiarity and meaningfulness, four negative words and four positive words were selected from each category. The valence of negative words and positive words significantly differed (*cognitive evaluation*: *t* = 9.52, *df* = 6, *p*<.01, *d* = 3.25; *affective reaction*: *t* = 17.13, *df* = 6, *p*<.01, *d* = 2.95; *behavioral tendency*: *t* = 10.37, *df* = 6, *p*<.01, *d* = 2.88). Informants who took part in the pilot study did not participate in the main experiment. We obtained the following stimuli for each category: mental illness (depression, obsessive-compulsive disorder, phobia disorder, and schizophrenia), positive words (*affective* SC-IAT: joyful, relaxed, pleasant, and cheerful; *behavioral* SC-IAT: approach, respect, care, and persuade; *cognitive* SC-IAT: admirable, competent, self-regard, and strong), and negative words (*affective* SC-IAT: boring, scared, nervous, and disgust; *behavioral* SC-IAT: reject, escape, despise, and avoid; *cognitive* SC-IAT: dangerous, abnormal, fragile, and pitiable). The three SC-IATs shared the same target categories but different attribute words. All participants completed the three SC-IATs in the order specified by a Latin square.

### Self-reported measures

Participants responded to two scales that measured their explicit attitudes toward mental illness. The first was the Social Distance Scale (SDS), which tested the desire to avoid people with mental illness [25], [26]. After reading a vignette about Li, a person with psychotic symptoms, five questions asked how willing (1 = *definitely unwilling*, 4 = *definitely willing*) the respondents would be to (1) move next door to Li, (2) spend an evening socializing with Li, (3) make friends with Li, (4) start working closely with Li, and (5) have Li marry into their families. These items of the SDS that were translated into Chinese were revised by psychology graduate students; Cronbach's alpha coefficient was 0.81 in the current study. The second scale was the Feeling Thermometer (FT), which was a visual analogue scale presented in the form of a thermometer from 1 (*extremely negative*) to 100 (*extremely positive*) [16], [27]. Respondents were asked to rate how positive or negative they found people with mental illness on the scale, with lower mean scores indicating more negative attitudes.

All data analyses were carried out by the SPSS 16.0 for Windows.

## Results

### The three SC-IATs of implicit stigma

For the SC-IAT, a scoring algorithm was modeled on the *D*-score algorithm used for the IAT data [16]. Karpinski and Steinman recommended that responses less than 350 ms be eliminated and that incorrect responses be replaced with the block mean plus an error penalty of 400 ms. First, the average response times of the compatible task (mental illness+negative words) were subtracted from the average response times of the incompatible task (mental illness+positive words). Second, this quantity was divided by the standard deviation of all correct response times within the compatible and incompatible tasks. This quotient is referred to as the *D*-score, with higher scores indicating stronger negative associations with mental illness.

Matched t-tests showed that the response times when mental illness was paired with negative words were faster relative to positive words in the three SC-IATs (*affective* SC-IAT: *t* = −149.05, *p*<.01; *behavioral* SC-IAT: *t* = −126.73, *p*<.01; *cognitive* SC-IAT: *t* = −133.28, *p*<.01; Table 2). The *D*-scores of the three SC-IATs were also computed according to the algorithm developed by Karpinski and Steinman. An independent-sample t-test indicated that the *D*-score of the *cognitive* SC-IAT was significantly above the zero point (*t* = 2.18, *p*<.05) and that of *affective* SC-IAT was marginally significant (*t* = 1.80, *p* = .078), but that of *behavioral* SC-IAT was not significant (*t* = 1.56, *p*>.05).

**Table 2:** Response time and D scores of three SC-IATs.

SC-IAT	Task	Response time(*M*, SD)	P-value^1^	D-score	P-value^2^
*Affective* SC-IAT	Compatible^3^ task	646.89, 264.12	<0.01	0.12	0.078
	Incompatible task	688.75, 383.57			
*Behavioral* SC-IAT	Compatible task	689.05, 289.95	<0.01	0.09	0.125
	Incompatible task	716.89, 367.35			
*Cognitive* SC-IAT	Compatible task	660.04, 253.04	<0.01	0.08	<0.05
	Incompatible task	688.96, 311.71			
The combined SC-IAT	Compatible task	665.36, 270.01	<0.01	0.10	<0.01
	Incompatible task	698.21, 355.68			

1 T test to response time between compatible and incompatible tasks;

2 T test to D-scores;

3 Compatible task is mental illness paired with negative words, and incompatible task is mental illness paired with positive words.

Although the three SC-IATs were developed basing on the same measurement (SC-IAT) and satisfied measurement equivalence, they are not able to evaluate implicit attitudes toward mental illness in Chinese samples separately. We thus merged the three SC-IATs into a combined SC-IAT to measure the overall attitudes to mental illness and performed matched t-test. The response times when mental illness was paired with negative words became faster relative to those with positive words (*t* = −234.311, *p*<0.01) after this adjustment, and the mean *D*-score was significantly above the zero point (*t* = 2.84, *p*<0.01). In addition, a frequency analysis found that 62.50% of the participants had *D*-scores above zero, indicating that these students tended to automatically associate mental illness with negative words rather than positive words.

### Self-reported attitudes toward mental illness

The score on the FT revealed a similarly neutral attitude (*M* = 51.02, SD = 20.03; *t* = .380, *p* = .705; Table 1). The scores of 60% of the participants were lower than 50 points. Table 1 also shows that participants scored below the 2.5 midpoint of the SDS, indicating some negative attitudes on the Social Distance Scale (*M* = 11.18, SD = 3.06; *t* = −2.731, *p*<.01). A frequency analysis showed that about 60% scored lower than the midpoints of SDS.

### Relationships between self-report and indirect measures

To examine the relationship between the explicit and implicit measures, the score of these measurements were transformed into *z*-scores before correlation analysis. The results of a bivariate correlation indicated that implicit attitudes toward mental illness, as indexed by the SC-IAT, were not related to explicit attitudes in a Chinese sample (all *p*>.05; Table 3). However, the correlations between the three separate and the combined SC-IATs were significant at the level of *r*>.42 (all *p*<.01; Table 3).

**Table 3:** Correlations between implicit and explicit measures among 56 members of the general public.

	Feeling Thermometer	Social Distance	*Cognitive* SC-IAT	*Affective* SC-IAT	*Behavioral* SC-IAT
Social Distance	0.23				
*Cognitive* SC-IAT	0.02	0.22			
*Affective* SC-IAT	0.14	0.09	0.42^**^		
*Behavioral* SC-IAT	0.08	0.03	0.47^**^	0.50^**^	
The combined SC-IAT	0.06	0.11	0.76^**^	0.74^**^	0.81^**^

*Notes*. all the measures were transformed into the *z*-scores before correlation analysis, and ‘**’ indicates *p*-value lower than 0.001.

**Figure 1:**
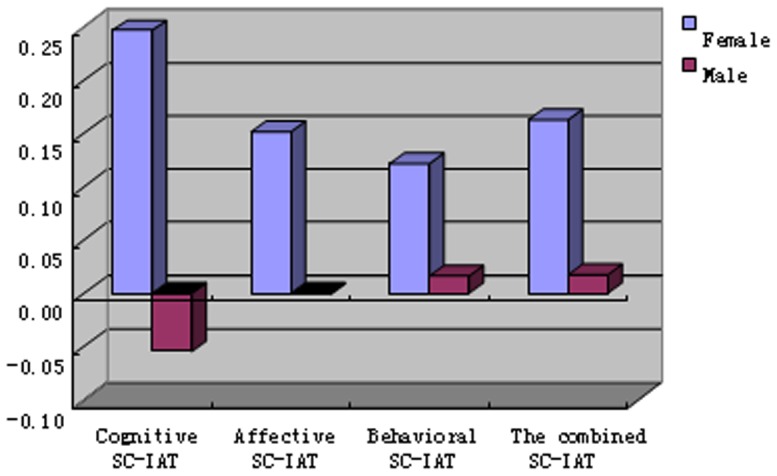
Gender differences in implicit stigma toward mental disorder.

**Figure 2:**
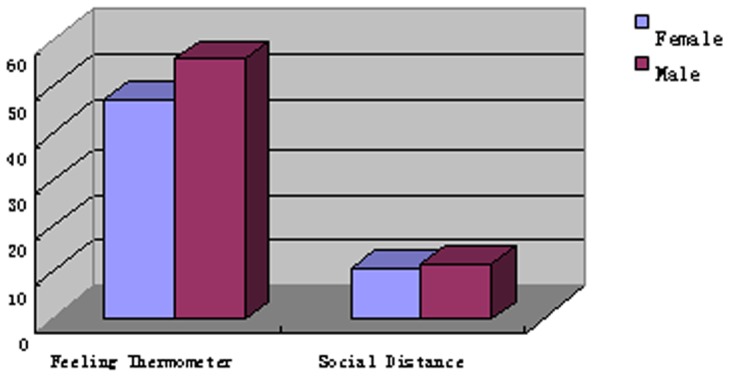
Gender differences in explicit stigma toward mental disorder.

We also explored differences in self-report and indirect measures by gender. Women were found to have more negative implicit attitudes toward mental illness than men (Figure 1). A MANOVA revealed that women scored significantly higher than men on the *D*-scores of the combined and *cognitive* SC-IATs (*F*(1, 54) = 4.327, *p*<.05, η*_p_* ^2^ = .137; *F*(1, 54) = 8.575, *p*<.01, η*_p_* ^2^ = .074), but not the *affective* and *behavioral* SC-IATs (*F*(1, 54) = 2.464, *p*>.05, η*_p_* ^2^ = .044 ; *F*(1, 54) = 1.122, *p*>.05, η*_p_* ^2^ = .020); no significant gender differences were found for the explicit stigma measures (all *p*>.05; Figure 2).

## Discussion

The present study examined key, theoretically derived components of implicit stigma toward mental illness with the SC-IAT. This model was supported by measurements of response times. The correlations between the three and the combined SC-IATs were significant at the level of *p*<0.05, confirming the convergent validity of the three-component model. The current findings suggested that automatically activated attitudes toward mental illness are multidimensional constructs, which include aspects that reflect negative cognitive evaluations, affective reactions and discriminatory tendencies.

A novel contribution of the current study was its investigation of these components by using three SC-IATs in a Chinese sample. Of particular interest was the observation that the results of the *cognitive* and *affective* SC-IATs were significant and reveal automatic cognitive evaluations and affective reactions, respectively, which is consistent with past research [6], [7], [10], but that of the *behavioral* SC-IAT was not. Overall, the implicit effect of negative evaluation was the strongest, followed by the effects of emotional reactions and, finally, discriminatory tendencies. We can thus speculate that the three components of implicit stigma toward mental illness may be hierarchical: the cognitive component is the basis of implicit prejudices, the emotional aspect plays a significant role in relation to adjustment and constraints, and the behavioral component has a direct relationship with explicit components [13]. Further examination of this theory is required in future research.

Another possible explanation for the comparatively weaker automatic discriminatory tendencies toward people with mental illness is the limited attribute words that do not perfectly reflect the behavioral aspects of implicit stigma. Especially, the process of balancing familiarity and meaningfulness may lead to miss some important information about automatic discriminatory tendencies. In addition, the weaker effect may be associated with the *D-*scores algorithm, which is only a reference by recommend Karpinski and Steinman, not the accurate standard. Indeed, the algorithm of IAT-based measures has been in the process of revision since the IAT was developed by Greenwald [16], [28], [29]. There are also mounting concerns that response times were faster when “mental illness” was paired with negative words than with positive words in the *behavioral* SC-IAT and that 60% of the participants showed negative associations with mental illness on this implicit measure. Accordingly, these findings provided at least partial support for the hypothesis that automatic attitudes are linked to discriminatory reactions based on the analysis of response times and frequencies. Future research should examine the levels of automatically activated discriminatory tendencies toward persons with mental illness.

General automatically activated attitudes toward mental illness were revealed by the combined SC-IAT in this Chinese sample, which corroborates with the findings of past research that used IAT or BIAT, indicating that implicit prejudice against mental illness exist relative to other target categories (e.g. physical illness and physical disability) [6], [7], [8], [10]. The current findings provide further evidence for implicit negative attitudes toward mental illness with the SC-IAT. Unfortunately, our study confirms a pessimistic view that, although overt prejudice and discrimination toward people with mental illness has become less socially acceptable, negative implicit attitudes toward them persist among Chinese young adults in a higher education setting. In addition, the frequency of overall attitudes toward mental illness was similar to the findings reported by Teachman et al [7], further suggesting that the majority of the participants had negative implicit attitudes toward mental illness.

Also in agreement with earlier findings [7], the discrepancy between the explicit scales, the FT and the SDS, indicated no definitive evidence of explicit negative stigma toward mental illness in the sample. These findings suggest that it is less socially acceptable among Chinese university students to express overt negative evaluations toward and discrimination against persons with mental illness. These tendencies may stem, in part, from efforts in recent years to improve public perception of people with mental illness. For example, all Chinese universities and colleges hold various activities to increase mental health knowledge, and to better students' mental health literacy through initiatives of the Ministry of Education, such as the College Students' Mental Health Day, which is held twice a year on May 25 and November 25. At the very least, such efforts draw attention to the issue of mental illness and may have stigma-reducing effects at the explicit level.

The attenuated correlation between explicit and implicit stigma in the current study was consistent with past researches [3], [7], [11]. This result may have been partly attributable to measurement or motivational variables [21], suggesting that implicit and explicit attitudes represent distinct constructs [29]. Furthermore, the low correlation between implicit and explicit measures points to the importance of evaluating both automatically activated and self-reported attitudes toward mental illness when considering stigma and anti-stigma. Implicit measures can tap attitudes toward mental illness that participants are either unwilling or unable to report explicitly. Hence, our findings encourage future study on implicit stigma toward mental illness to address the shortcomings of relying upon self-report measures.

We also examined gender differences in mental illness stigma. Interestingly, unlike the previous studies that found no sex differences in implicit attitudes toward mental illness [7], our results indicated that women had significantly more negative overall implicit attitudes, especially negative cognition and beliefs, toward mental illness than men in our sample. Although there was no significant difference in the *D-*scores of *affective* and *behavioral* SC-IATs, women had more negative scores than men on automatically activated affective reactions and behavioral tendencies. These findings indicate that men and women have different implicit beliefs and expressed different automatic feelings and behavioral tendencies toward people with mental illness. There are a number of possible explanations for these gender differences, including the possibility that men tend to be more assertive, risk-taking and dominance-seeking than women. At the same time, women are generally more anxiety, cautious and shy than men [30], [31]. These traits are presumably associated with the gender differences in implicit stigma. Therefore, men would have less inhibition about interacting with people with mental illness and consequently have less negative implicit attitudes toward them than women. These findings also correspond with the gender differences in coping styles, where women are more likely to use more passive, emotional and avoiding coping styles than men [32]. These differences in coping styles may contribute to gender differences in reactions and attitudes to people with mental illness. In summary, our findings suggest that gender appears to be a factor in implicit stigma toward mental illness and its intervention.

Unlike gender differences in implicit stigma, there were no sex differences in explicit stigma measures. Although a review revealed that women acted in a much more benign and favorable way toward people with mental illness than men did [33], recent researches, including the current study, found no significant gender differences in explicit stigma toward mental illness [7]. One possible explanation for these findings is that overt prejudice toward mental illness has become less acceptable in modern society. This social desirability may lead both women and men to endorse the same attitudes toward mental illness at the explicit level. This is particularly true in Chinese culture, where harmonious interactions between people serve as the basis of society [34]. Women and men do not readily express their negative attitudes toward others; such exhibitions are usually viewed as recklessness and undignified. Accordingly, it is not surprising that the women and men in our sample did not have significant negative attitudes against mental illness at the explicit level. It must be said, however, that no consistent relationships were found between mental illness stigma and gender in previous reviews [1]. Therefore, further research is needed to explore this difference in Chinese participants.

Despite its implications, select limitations of the current study must be acknowledged. First, our data was correlational in nature and did not allow us to draw conclusions about causality. Second, we focused on key aspects of implicit stigma toward mental illness with a limited number of attribute words; therefore other aspects of implicit stigma (e.g., “loss of face,” “shame,” and “destructive”) warrant inclusion in future studies. Third, we assessed attitudes toward mental illness among undergraduate students, a group that is not representative of the general public in China, most notably in terms of age and sociodemograhic variables. Moreover, the results of our study may be hampered by family members' history of mental illness, the single approach in recruiting participants, race, and religion. Fourth, our study examined attitudes toward mental illness in general, and did not differentiate between diagnoses such as depression and schizophrenia. Finally, several implicit measures have been developed to assess evaluative associations with a single target, for example, the Extrinsic Affective Simon Task, the go/no-go Association Task, and priming-based measures [17], [18], [35]. Supporting evidence from these implicit measures would bolster the convergent validity of this study. Thus, future research should use other methods to examine how different labels and attribute words affect automatically-activated negative associations.

In conclusion, our research provided further evidence that mental illness stigma can be automatically activated. We also found further support for the three components of implicit stigma toward mental illness as determined by the SC-IAT. Nevertheless, future studies should further investigate the discriminatory tendencies dimension of implicit stigma toward mental illness. Despite recent efforts to reduce stigma, negative attitudes against mental illness persist at an implicit level among young adult students in China. It is thus important to consider the property and components of implicit stigma to improve the respect and care of people toward persons with mental illness and to reduce the negative effects of stigma. Furthermore, initiatives to reduce stigma should consider how to best integrate explicit and implicit measures in order to track changes in stigma attitudes following interventions.
